# How does HbA1c predict mortality and readmission in patients with heart failure? A protocol for systematic review and meta-analysis

**DOI:** 10.1186/s13643-023-02179-4

**Published:** 2023-03-10

**Authors:** Jun-Peng Xu, Rui-Xiang Zeng, Xiao-Yi Mai, Wen-Jun Pan, Yu-Zhuo Zhang, Min-Zhou Zhang

**Affiliations:** 1 The Second Clinical College of Guangzhou University of Chinese Medicine, Guangzhou, 510405 China; 2 The Guangzhou University of Chinese Medicine, Guangzhou, 510405 China; 3 Department of Critical Care Medicine, Guangdong Provincial Hospital of Chinese Medicine, 111 Dade Road, Yuexiu District, Guangzhou, 510120 Guangdong Province China

**Keywords:** HbA1c, Heart failure, Mortality, Readmission, Systematic review and meta-analysis, Protocol

## Abstract

**Background:**

Accumulating evidence suggests that HbA1c levels, a common clinical indicator of chronic glucose metabolism over the preceding 2–3 months, are independent risk factors for cardiovascular disease, including heart failure. However, conflicting evidence obscures clear cutoffs of HbA1c levels in various heart failure populations. The aim of this review is to assess the possible predictive value and optimal range of HbA1c on mortality and readmission in patients with heart failure.

**Methods:**

A systematic and comprehensive search will be performed using PubMed, Embase, CINAHL, Scopus, and the Cochrane Library databases before December 2022 to identify relevant studies. All-cause mortality is the prespecified primary endpoint. Cardiovascular death and heart failure readmission are secondary endpoints of interest. We will only include prospective and retrospective cohort studies and place no restrictions on the language, race, region, or publication period. The ROBINS-I tool will be used to assess the quality of each included research. If there were sufficient studies, we will conduct a meta-analysis with pooled relative risks and corresponding 95% confidence intervals to evaluate the possible predictive value of HbA1c for mortality and readmission. Otherwise, we will undertake a narrative synthesis. Heterogeneity and publication bias will be assessed. If heterogeneity was significant among included studies, a sensitivity analysis or subgroup analysis will be used to explore the source of heterogeneity, such as diverse types of heart failure or patients with diabetes and non-diabetes. Additionally, we will conduct meta-regression to examine the time-effect and treatment-effect modifiers on all-cause mortality compared between different quantile of HbA1c levels. Finally, a restricted cubic spline model may be used to explore the dose-response relationship between HbA1c and adverse outcomes.

**Discussion:**

This planned analysis is anticipated to identify the predictive value of HbA1c for mortality and readmission in patients with heart failure. Improved understanding of different HbA1c levels and their specific effect on diverse types of heart failure or patients with diabetes and non-diabetes is expected to be figured out. Importantly, a dose-response relationship or optimal range of HbA1c will be determined to instruct clinicians and patients.

**Systematic review registration:**

PROSPERO registration details: CRD42021276067

**Supplementary Information:**

The online version contains supplementary material available at 10.1186/s13643-023-02179-4.

## Background

Heart failure is a major cardiovascular complication of diabetes mellitus (DM), and they beget each other, while the links between the 2 conditions are not fully elucidated. In the heart failure cohort, including heart failure with reduced and preserved left ventricular ejection fraction (LVEF), the prevalence of DM ranges from 10 to 47% [1, 2]. In particular, the prevalence of DM is more than 40% in newly hospitalized patients with heart failure [3]. In addition, robust evidence suggests that DM is associated with a nearly 2- to 4-fold increase in the risk of incident heart failure, even after adjustment for other cardiovascular risk factors [4]. Furthermore, poor glycemic control is related to an increased incident of heart failure: for a 1% increase in HbA1c, the risk of incident heart failure increases by 15% [5]. Thus, elevated HbA1c might be closely related to incidence and prognosis of heart failure patients with DM. However, the question how does HbA1c predict mortality and readmission in patients with heart failure has not been figured out. Although current guidelines recommend a target level of HbA1c < 7% in type 2 DM patients [6], some studies proved that HbA1c could independently predict mortality with U-shaped relationship, and the lowest mortality HbA1c range was 6.5–7.9% [7, 8], while the other showed a direct relationship with HbA1c < 6.5% or inverse relationship with HbA1c ≥ 8.7% [9, 10]. Furthermore, Lejeune et al [11]. demonstrated HbA1c > 7% was protective for patients following heart failure with preserved ejection fraction, whereas some studies indicated HbA1c > 7% was associated with higher rate of adverse outcomes, including mortality, in patients following heart failure with reduced ejection fraction [12, 13]. These mean the effect of different HbA1c levels on adverse outcomes may be various in response to the types of heart failure. In spite of accumulating evidence, there are no further pooled analysis to figure out what HbA1c levels should be controlled for heart failure patients with pre-DM or non-DM. Thus, we aim to present a qualitative and quantitative analysis from observational cohort studies with conclusive evidence and summary existing information on the relationship of HbA1c levels with mortality and readmission in patients with heart failure.

## Method and analysis

### Review design

Reviewing and synthesizing the observational research will address a critical gap in the evidence on the potential association between HbA1c exposure and mortality and readmission in patients with heart failure. Furthermore, observational research typically allows for longer follow-up time with fewer ethical concerns and can examine the outcomes of a broader participant group under naturalistic circumstances. Thus, we will include prospective and retrospective observational cohort studies defined according to the Cochrane study design guide [14] and will perform meta-analyses in cases of sufficient available data. Otherwise, we will undertake a systematic review. The study has been registered to the International Prospective Register of Systematic Reviews (PROSPERO registration number: CRD42021276067). This protocol adheres to the latest Preferred Reporting Items for Systematic Reviews and Meta-analysis (PRISMA) statement (see Additional file 1) [15].

### Eligibility criteria

The inclusion and exclusion criteria are defined according to the Population of interest, Exposure, Comparator and Outcome (PECO) statements and outlined in Table 1. Our population of interest will be adult participants with all-type heart failure regardless of glucose metabolic state. In accordance with the rationale and objective described earlier, exposure, comparator, and outcomes were various HbA1c levels and the related risk of all-cause mortality, cardiovascular mortality, and heart failure readmission. Diagnosis and classification of heart failure are following the guidelines: heart failure with reduced (LVEF < 40%), mid-range (LVEF: 40 to < 50%), or preserved ejection fraction (LVEF ≥ 50%) [16]. Definition of different glucose metabolic state according to HbA1c using the International Diabetes Expert Committee criteria: (1) without DM, < 6.5%, and with DM, ≥ 6.5% [17].

**Table 1:** Summary of inclusion and exclusion criteria

	Inclusion criteria	Exclusion criteria
Participants	Adult patients with heart failure regardless of glucose metabolic state	Patients with acute coronary syndrome or cardiovascular revascularization less than 3 months
Exposure	Reporting HbA1c as continuous and/or categorical variables	Other
Comparator	Various categorical HbA1c levels	NA
Outcomes	All-cause mortality, cardiovascular death and heart failure readmission	NA
Study design	Prospective and retrospective cohort	Case-control, cross-sectional, randomized controlled trials, case report, case series, and any other non-relevant studies

### Literature review

A literature search will be conducted using PubMed, Embase, CINAHL, Scopus, and the Cochrane Library databases from the date of inception until December 2022. The following Mesh terms will be used in search: heart failure, systolic heart failure, and diastolic heart failure combined with glycated hemoglobin a. Corresponding free texts are as follows: cardiac failure, heart decompensation, congestive heart failure, right-sided heart failure, myocardial failure, left-sided heart failure, hemoglobin a1c, hemoglobin a1c, HbA1c, glycated hemoglobin, glycated hemoglobin, glycosylated hemoglobin, glycosylated hemoglobin, glycohemoglobin a, or glycohemoglobin a. The search strategy for PubMed is presented in Additional file 2. Additional eligible studies from previous version of established reviews and the reference lists of retrieved articles will be manually reviewed, in cases of using other databases simultaneously. Search results will be exported, and duplicates will be removed using software NoteExpress.

### Study screening and selection

**Fig. 1:**
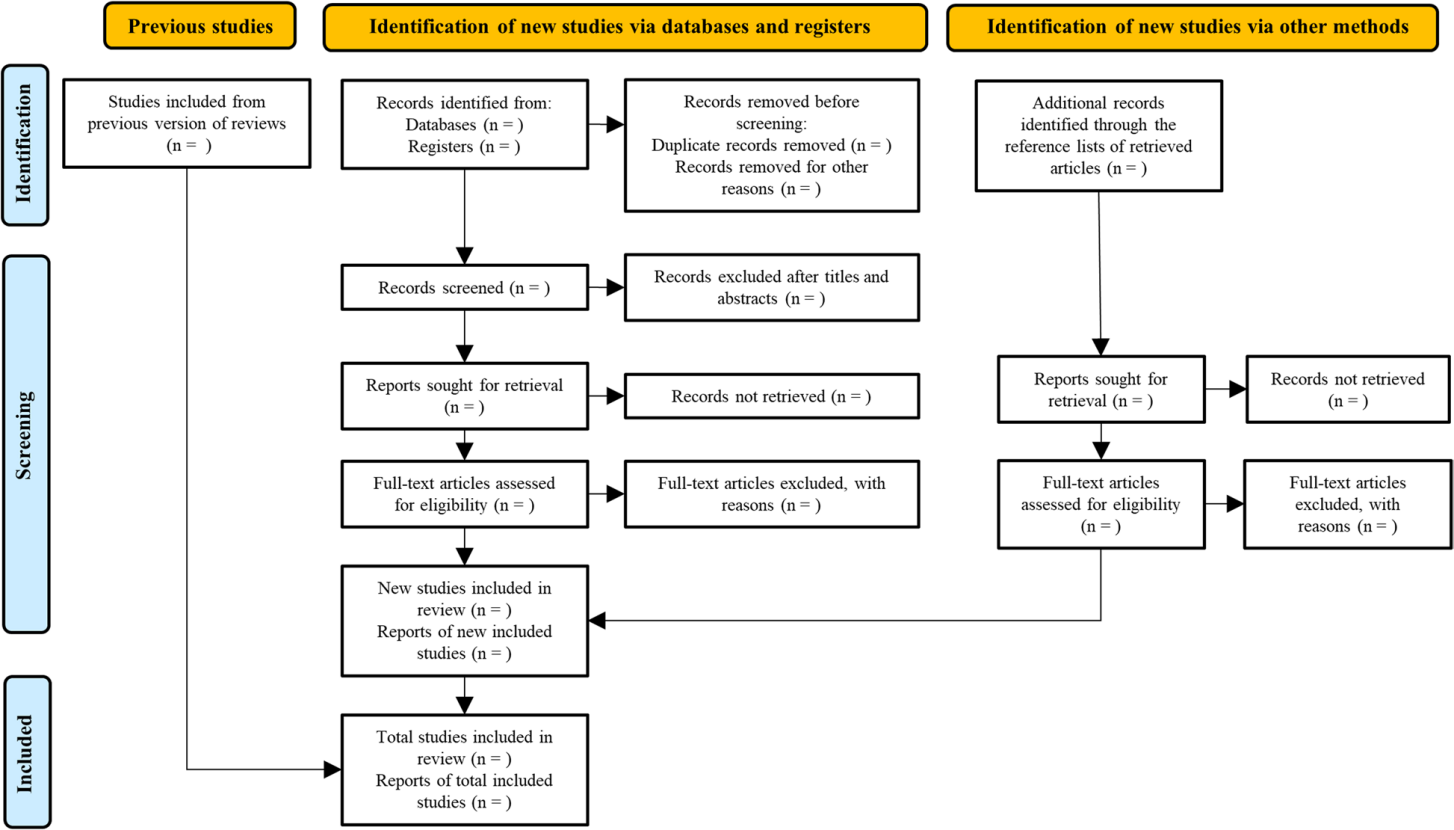
Literature search PRISMA (Preferred Reporting Items for Systematic Reviews and Meta-analysis) consort diagram

According to the inclusion and exclusion criteria, the process of identifying, screening, and including of those studies is shown in the PRISMA flow chart (Fig. 1). All titles and abstracts will be independently reviewed by two authors to determine appropriate studies included. Similarly, other two independent authors will review full texts and compare these against predefined eligibility criteria. A senior author will resolve any conflicts regarding inclusion or exclusion of articles through discussion. All authors hold expertise relevant to the review subject matter. An excluded study list will record articles that did not meet inclusion criteria, but that may provide information of interest to readers.

### Data extraction and risk of bias assessment

We will extract data of interest from all included studies using revised versions of a previously piloted data extraction form (Additional file 3). All review screening, bias assessment, and data extraction will be managed on the excel software. Two authors will blindingly extract the data on first author’s name, publication year, age range and/or mean age (years), number of participants, mean follow-up duration, the types of heart failure and glucose metabolic state, HbA1c exposure levels, and RRs and their 95% CIs as well as event numbers for each exposure category (Table 2). The quality of the studies will be also assessed by the two authors and using the Risk Of Bias In Non-randomized Studies-of Interventions (ROBINS-I), a validated quality assessment instrument for observational cohort studies [18]. It encompasses 7 domains: confounding, selection of participants, classification of the intervention, deviation from the intended intervention, missing data, measurement of outcomes, and selection of the reported results. Each domain will be judged as either low, moderate, serious, or critical risk of bias or no information available. If agreement in bias assessment cannot be reached, the dispute will be resolved with the help of other two investigators. Any discrepancies will be resolved through discussion under supervision of a senior author. Publication bias will be assessed using funnel plots’ asymmetry and tested with Egger’s asymmetry test and Begg’s test (*p* < 0.05) when there are at least 10 studies.

**Table 2:** Characteristics of included studies

First author	Year	Number screened	Age at screening	Men (%)	Follow-up	HF types	Glucose metabolic state	Country/continent	Quality score
Author name	Year of publication	Number of participants	Mean age of participants	Men (%)	Mean follow-up time	HFpEF, HFmrEF, and HFrEF	With DM and without DM (or including without known DM)	Country/continent	ROBINS-I scores

*HF* heart failure, *HFpEF* heart failure with preserved ejection fraction, *HFmrEF* heart failure with mid-range ejection fraction, *HFrEF* heart failure with reduced ejection fraction, *DM* diabetes mellitus

### Data synthesis

Evidence regarding the association with HbA1c exposure of the three outcomes, including all-cause mortality, cardiovascular mortality, and heart failure readmission, will be reported according to the latest PRISMA criteria [15] and satisfy the Meta-analysis Of Observational Studies in Epidemiology (MOOSE) for Meta-analyses of Observational Studies [19]. The predictive value of HbA1c was measured in the form of a relative risk [risk ratio (RR), odds ratio (OR) or hazard ratio (HR)] and their 95% confidence interval (CI) or the event number and total number of each group. If there are sufficient studies, we will conduct meta-analysis with pooled relative risks and corresponding 95% CI to assess the possible predictive value and optimal range of HbA1c on mortality and readmission. Otherwise, we will undertake a narrative synthesis, or in cases of primary studies with unavailable data, and extremely high heterogeneity between their populations and design.

### Statistical analysis

We will calculate the average RRs for various categorical HbA1c levels using a fixed-effects or random-effects model. If RRs and their 95% CI cannot be available in the papers, the unadjusted ones will be calculated through original data published in the studies or contacting the studying authors. The *I*^2^ statistic and Cochrane’s *Q* test are used to estimate the heterogeneity. The *I*^2^ value is from 0 to 25% and 26 to 50%, indicating little and acceptable moderate heterogeneity, respectively. If heterogeneity is significant among studies (*I*^2^ > 50%, *p* < 0.1), a sensitivity analysis or subgroup analysis will be used to explore the source of heterogeneity. The strength of the body of evidence will be evaluated using the Grading of Recommendations Assessment, Development and Evaluation (GRADE) tool [20]. All analysis will be conducted with Stata 12.0.

### Subgroup analyses and meta-regression

First, given different cardiac and glucose metabolic dysfunction will influence the risk of survival and readmission, we will abstract relevant data and conduct subgroup analyses to validate the distribution of the most at-risk population if possible. Second, subgroup analyses will be performed to evaluate the specific effect of different HbA1c levels on different types of heart failure and patients with DM or non-DM. Furthermore, the meta-regression will be performed based on time-effect and treatment-effect, including follow-up duration, anti-heart failure, and anti-diabetic drugs, because these may be major factors influencing the primary and secondary outcomes and contribute to high or complete inter-research variance [21]. The main reason may be attenuation of medicine adherence over time [21]. Finally, a dose-response relationship or optimal range of HbA1c to predict adverse outcomes in these population will be determined if possible.

## Discussion

The predictive value of HbA1c levels as a prognostic marker for mortality and readmission is uncertainty in spite of numerous studies with conclusive evidence. Patients with DM represent a large portion of adults with chronic heart failure. Also, patients hospitalized with acute heart failure are always concomitant with hyperglycemia and high levels of HbA1c in cases of no previous diagnosis of DM. Besides, a narrative review on this topic have been previously published and showed that there was certain variation between included studies [22]. Although a previous protocol also studied the predictive effect of HbA1c on the cardiovascular events and mortality [23], it did not either entirely analyze the possibly various effect on different types of population, such as diverse types of heart failure, or explore the dose-response relationship between HbA1c and adverse outcomes. Therefore, it is necessary to conduct a systematic review to figure out what HbA1c levels and how to predict mortality and readmission in patients with heart failure, including the specific effect on diverse types of heart failure or patients with diabetes and non-diabetes.

We anticipate some possible limitations to our systematic review and meta-analysis. Firstly, there may be some problems in primary studies, such as publication bias, information bias, poor statistical analyses, and inadequate reporting of different levels of HbA1c. Second, the follow-up duration of observational studies may be longer but always inconsistent. Third, heart failure itself may also contribute to increased risk of mortality and readmission. However, it is vital to pool all available information on this issue. In order to overcome potential limitations, we will strictly follow the MOOSE and PRISMA guidelines.

## Supplementary Information

**Additional file 1.**

**Additional file 2.**

**Additional file 3.**

## Data Availability

The data used in this study will be extracted from published literature identified through PubMed, Embase, CINAHL, Scopus, and the Cochrane Library databases.

## Abbreviations

HbA1c: Hemoglobin a1c
DM: Diabetes mellitus
LVEF: left ventricular ejection fraction
RR: Risk ratio
OR: Odds ratio
HR: Hazard ratio
CI: Confidence interval
ROBINS-I: Risk Of Bias In Non-randomized Studies-of Interventions
PRISMA: Preferred Reporting Items for Systematic Reviews and Meta-analysis
MOOSE: Meta-analysis of Observational Studies in Epidemiology
