# Supplementary material for: How does HbA1c predict mortality and readmission in patients with heart failure? A protocol for systematic review and meta-analysis
Source: Syst Rev. 2023 Mar 10;12:35. doi: 10.1186/s13643-023-02179-4 (PMC10007851; doi:10.1186/s13643-023-02179-4)
Supplement: Supplementary file 2 — Additional file 2. [file 13643_2023_2179_MOESM2_ESM.pdf]

Search number	Query
9	((((((((((((((((((((((((((heart failure[Title/Abstract]) OR (Cardiac Failure[Title/Abstract])) OR (Heart Decompensation[Title/Abstract])) OR (Decompensation, Heart[Title/Abstract])) OR (Heart Failure, Right-Sided[Title/Abstract])) OR (Heart Failure, Right Sided[Title/Abstract])) OR (Right-Sided Heart Failure[Title/Abstract])) OR (Right Sided Heart Failure[Title/Abstract])) OR (Myocardial Failure[Title/Abstract])) OR (Congestive Heart Failure[Title/Abstract])) OR (Heart Failure, Congestive[Title/Abstract])) OR (Heart Failure, Left-Sided[Title/Abstract])) OR (Heart Failure, Left Sided[Title/Abstract])) OR (Left-Sided Heart Failure[Title/Abstract])) OR (Left Sided Heart Failure[Title/Abstract])) OR (systolic heart failure[Title/Abstract])) OR (Heart Failures, Systolic[Title/Abstract])) OR (Systolic Heart Failures[Title/Abstract])) OR (Systolic Heart Failure[Title/Abstract])) OR (diastolic heart failure[Title/Abstract])) OR (Diastolic Heart Failures[Title/Abstract])) OR (Heart Failures, Diastolic[Title/Abstract])) OR (Diastolic Heart Failure[Title/Abstract])) OR ("Heart Failure, Diastolic"[Mesh])) OR ("Heart Failure, Systolic"[Mesh])) OR ("Heart Failure"[Mesh])) AND ((hemoglobin a1c[Title/Abstract]) OR (Hemoglobin A, Glycated[Title/Abstract])) OR (Hb A1a+b[Title/Abstract])) OR (Hb A1c[Title/Abstract])) OR (HbA1[Title/Abstract])) OR (Glycosylated Hemoglobin A[Title/Abstract])) OR (Hemoglobin A, Glycosylated[Title/Abstract])) OR (Hb A1[Title/Abstract])) OR (Glycohemoglobin A[Title/Abstract])) OR (Hemoglobin A(1)[Title/Abstract])) OR (Hb A1a-2[Title/Abstract])) OR (Hemoglobin, Glycated A1a-2[Title/Abstract])) OR (A1a-2 Hemoglobin, Glycated[Title/Abstract])) OR (Glycated A1a-2 Hemoglobin[Title/Abstract])) OR (Hemoglobin, Glycated A1a 2[Title/Abstract])) OR (Hemoglobin, Glycosylated A1a-1[Title/Abstract])) OR (A1a-1 Hemoglobin, Glycosylated[Title/Abstract])) OR (Glycosylated A1a-1 Hemoglobin[Title/Abstract])) OR (Hemoglobin, Glycosylated A1a 1[Title/Abstract])) OR (Hb A1a-1[Title/Abstract])) OR (Hemoglobin, Glycated A1b[Title/Abstract])) OR (A1b Hemoglobin, Glycated[Title/Abstract])) OR (Glycated A1b Hemoglobin[Title/Abstract])) OR (Hb A1b[Title/Abstract])) OR (Hemoglobin, Glycosylated A1b[Title/Abstract])) OR (A1b Hemoglobin, Glycosylated[Title/Abstract])) OR (Glycosylated A1b Hemoglobin[Title/Abstract])) OR (Glycated Hemoglobin A1c[Title/Abstract])) OR (Hemoglobin A1c, Glycated[Title/Abstract])) OR (Glycosylated Hemoglobin A1c[Title/Abstract])) OR (Hemoglobin A1c, Glycosylated[Title/Abstract])) OR (Glycated Hemoglobins[Title/Abstract])) OR (Hemoglobins, Glycated[Title/Abstract])) OR (Hemoglobin, Glycosylated[Title/Abstract])) OR (Glycosylated Hemoglobin[Title/Abstract])) OR (Glycated Hemoglobin[Title/Abstract])) OR (Hemoglobin, Glycated[Title/Abstract])) OR (HbA1c[Title/Abstract])) OR (glycated hemoglobin[Title/Abstract])) OR (glycosylated hemoglobin[Title/Abstract])) OR (haemoglobin a1c[Title/Abstract])) OR (glycated haemoglobin[Title/Abstract])) OR (glycosylated haemoglobin[Title/Abstract])) OR (glycohemoglobin a[Title/Abstract])) OR (glycohaemoglobin a[Title/Abstract])) OR ("Glycated Hemoglobin A"[Mesh]))
8	(((hemoglobin a1c[Title/Abstract]) OR (Hemoglobin A, Glycated[Title/Abstract])) OR (Hb A1a+b[Title/Abstract])) OR (Hb A1c[Title/Abstract])) OR (HbA1[Title/Abstract])) OR (Glycosylated Hemoglobin A[Title/Abstract])) OR (Hemoglobin A, Glycosylated[Title/Abstract])) OR (Hb A1[Title/Abstract])) OR (Glycohemoglobin A[Title/Abstract])) OR (Hemoglobin A(1)[Title/Abstract])) OR (Hb A1a-2[Title/Abstract])) OR (Hemoglobin, Glycated A1a-2[Title/Abstract])) OR (A1a-2 Hemoglobin, Glycated[Title/Abstract])) OR (Glycated A1a-2 Hemoglobin[Title/Abstract])) OR (Hemoglobin, Glycated A1a 2[Title/Abstract])) OR (Hemoglobin, Glycosylated A1a-1[Title/Abstract])) OR (A1a-1 Hemoglobin, Glycosylated[Title/Abstract])) OR (Glycosylated A1a-1 Hemoglobin[Title/Abstract])) OR (Hemoglobin, Glycosylated A1a 1[Title/Abstract])) OR (Hb A1a-1[Title/Abstract])) OR (Hemoglobin, Glycated A1b[Title/Abstract])) OR (A1b Hemoglobin, Glycated[Title/Abstract])) OR (Glycated A1b Hemoglobin[Title/Abstract])) OR (Hb A1b[Title/Abstract])) OR (Hemoglobin, Glycosylated A1b[Title/Abstract])) OR (A1b Hemoglobin, Glycosylated[Title/Abstract])) OR (Glycosylated A1b Hemoglobin[Title/Abstract])) OR (Glycated Hemoglobin A1c[Title/Abstract])) OR (Hemoglobin A1c, Glycated[Title/Abstract])) OR (Glycosylated Hemoglobin A1c[Title/Abstract])) OR (Hemoglobin A1c, Glycosylated[Title/Abstract])) OR (Glycated Hemoglobins[Title/Abstract])) OR (Hemoglobins, Glycated[Title/Abstract])) OR (Hemoglobin, Glycosylated[Title/Abstract])) OR (Glycosylated Hemoglobin[Title/Abstract])) OR (Glycated Hemoglobin[Title/Abstract])) OR (Hemoglobin, Glycated[Title/Abstract])) OR (HbA1c[Title/Abstract])) OR (glycated hemoglobin[Title/Abstract])) OR (glycosylated hemoglobin[Title/Abstract])) OR (haemoglobin a1c[Title/Abstract])) OR (glycated haemoglobin[Title/Abstract])) OR (glycosylated haemoglobin[Title/Abstract])) OR (glycohemoglobin a[Title/Abstract])) OR (glycohaemoglobin a[Title/Abstract])) OR ("Glycated Hemoglobin A"[Mesh])
7	((hemoglobin a1c[Title/Abstract]) OR (Hemoglobin A, Glycated[Title/Abstract])) OR (Hb A1a+b[Title/Abstract])) OR (Hb A1c[Title/Abstract])) OR (HbA1[Title/Abstract])) OR (Glycosylated Hemoglobin A[Title/Abstract])) OR (Hemoglobin A, Glycosylated[Title/Abstract])) OR (Hb A1[Title/Abstract])) OR (Glycohemoglobin A[Title/Abstract])) OR (Hemoglobin A(1)[Title/Abstract])) OR (Hb A1a-2[Title/Abstract])) OR (Hemoglobin, Glycated A1a-2[Title/Abstract])) OR (A1a-2 Hemoglobin, Glycated[Title/Abstract])) OR (Glycated A1a-2 Hemoglobin[Title/Abstract])) OR (Hemoglobin, Glycated A1a 2[Title/Abstract])) OR (Hemoglobin, Glycosylated A1a-1[Title/Abstract])) OR (A1a-1 Hemoglobin, Glycosylated[Title/Abstract])) OR (Glycosylated A1a-1 Hemoglobin[Title/Abstract])) OR (Hemoglobin, Glycosylated A1a 1[Title/Abstract])) OR (Hb A1a-1[Title/Abstract])) OR (Hemoglobin, Glycated A1b[Title/Abstract])) OR (A1b Hemoglobin, Glycated[Title/Abstract])) OR (Glycated A1b Hemoglobin[Title/Abstract])) OR (Hb A1b[Title/Abstract])) OR (Hemoglobin, Glycosylated A1b[Title/Abstract])) OR (A1b Hemoglobin, Glycosylated[Title/Abstract])) OR (Glycosylated A1b Hemoglobin[Title/Abstract])) OR (Glycated Hemoglobin A1c[Title/Abstract])) OR (Hemoglobin A1c, Glycated[Title/Abstract])) OR (Glycosylated Hemoglobin A1c[Title/Abstract])) OR (Hemoglobin A1c, Glycosylated[Title/Abstract])) OR (Glycated Hemoglobins[Title/Abstract])) OR (Hemoglobins, Glycated[Title/Abstract])) OR (Hemoglobin, Glycosylated[Title/Abstract])) OR (Glycosylated Hemoglobin[Title/Abstract])) OR (Glycated Hemoglobin[Title/Abstract])) OR (Hemoglobin, Glycated[Title/Abstract])) OR (HbA1c[Title/Abstract])) OR (glycated hemoglobin[Title/Abstract])) OR (glycosylated hemoglobin[Title/Abstract])) OR (haemoglobin a1c[Title/Abstract])) OR (glycated haemoglobin[Title/Abstract])) OR (glycosylated haemoglobin[Title/Abstract])) OR (glycohemoglobin a[Title/Abstract])) OR (glycohaemoglobin a[Title/Abstract])
6	"Glycated Hemoglobin A"[Mesh]

5	((((((((((((((((((((((((((heart failure[Title/Abstract]) OR (Cardiac Failure[Title/Abstract])) OR (Heart Decompensation[Title/Abstract])) OR (Decompensation, Heart[Title/Abstract])) OR (Heart Failure, Right-Sided[Title/Abstract])) OR (Heart Failure, Right Sided[Title/Abstract])) OR (Right-Sided Heart Failure[Title/Abstract])) OR (Right Sided Heart Failure[Title/Abstract])) OR (Myocardial Failure[Title/Abstract])) OR (Congestive Heart Failure[Title/Abstract])) OR (Heart Failure, Congestive[Title/Abstract])) OR (Heart Failure, Left-Sided[Title/Abstract])) OR (Heart Failure, Left Sided[Title/Abstract])) OR (Left-Sided Heart Failure[Title/Abstract])) OR (Left Sided Heart Failure[Title/Abstract])) OR (systolic heart failure[Title/Abstract])) OR (Heart Failures, Systolic[Title/Abstract])) OR (Systolic Heart Failures[Title/Abstract])) OR (Systolic Heart Failure[Title/Abstract])) OR (diastolic heart failure[Title/Abstract])) OR (Diastolic Heart Failures[Title/Abstract])) OR (Heart Failures, Diastolic[Title/Abstract])) OR (Diastolic Heart Failure[Title/Abstract])) OR ("Heart Failure, Diastolic"[Mesh])) OR ("Heart Failure, Systolic"[Mesh])) OR ("Heart Failure"[Mesh])
4	((((((((((((((((((((((heart failure[Title/Abstract]) OR (Cardiac Failure[Title/Abstract])) OR (Heart Decompensation[Title/Abstract])) OR (Decompensation, Heart[Title/Abstract])) OR (Heart Failure, Right-Sided[Title/Abstract])) OR (Heart Failure, Right Sided[Title/Abstract])) OR (Right-Sided Heart Failure[Title/Abstract])) OR (Right Sided Heart Failure[Title/Abstract])) OR (Myocardial Failure[Title/Abstract])) OR (Congestive Heart Failure[Title/Abstract])) OR (Heart Failure, Congestive[Title/Abstract])) OR (Heart Failure, Left-Sided[Title/Abstract])) OR (Heart Failure, Left Sided[Title/Abstract])) OR (Left-Sided Heart Failure[Title/Abstract])) OR (Left Sided Heart Failure[Title/Abstract])) OR (systolic heart failure[Title/Abstract])) OR (Heart Failures, Systolic[Title/Abstract])) OR (Systolic Heart Failures[Title/Abstract])) OR (Systolic Heart Failure[Title/Abstract])) OR (diastolic heart failure[Title/Abstract])) OR (Diastolic Heart Failures[Title/Abstract])) OR (Heart Failures, Diastolic[Title/Abstract])) OR (Diastolic Heart Failure[Title/Abstract])
3	"Heart Failure, Diastolic"[Mesh]
2	"Heart Failure, Systolic"[Mesh]
1	"Heart Failure"[Mesh]
